# Stability and Activity of the Hyperglycosylated Human Interferon-β R27T Variant

**DOI:** 10.1038/s41598-020-65495-y

**Published:** 2020-05-21

**Authors:** Kyoung Song, Dae Bong Moon, Na Young Kim, Young Kee Shin

**Affiliations:** 1 R&DB Center, LOGONE Bio-convergence Research Foundation, Seoul, Republic of Korea; 2 IFEZ Bio Analysis Center, Binex Co., Ltd., Incheon, Republic of Korea; 3 ABION Inc., R&D Center, Seoul, Republic of Korea; 4 Research Institute of Pharmaceutical Sciences, College of Pharmacy, Seoul National University, Seoul, Republic of Korea; 5 Molecular Medicine and Biopharmaceutical Sciences, Graduate School of Convergence Science and Technology, Seoul National University, Seoul, Republic of Korea; 6 Bio-MAX/N-Bio, Seoul National University, Seoul, Republic of Korea

**Keywords:** Biotechnology, Mass spectrometry

## Abstract

A hyperglycosylated recombinant human interferon-β (rhIFN-β) R27T mutant was established to improve relapsing-remitting multiple sclerosis (RRMS) in our previous study. We focused on the stability of the R27T mutant throughout its production lifetime, including culture, purification, and storage before formulation prior to clinical use. Herein, we address the stability of this protein during optimized culture and purification processes. Additionally, we employed artificial stress conditions during culture and purification to characterize R27T instability. Although, among total R27T, relative native R27T ratio displayed transiently low even under optimized production process, the ratio was recovered by the end of the overall production process, suggesting that culture and purification processes are optimized. Artificial stress during culture and purification processes resulted in degradation of R27T acidic and basic variants, and mismatched disulfide bonds in no-aggregated forms as well as in the aggregated form. The presence of disulfide bond exchange without aggregation in the unfolded/misfolded state could be a novel finding for rhIFN-β products. The results provide meaningful information for the comprehensive evaluation of the stability of the R27T variant.

## Introduction

Maintaining protein stability is a critical issue throughout all development, culture, refolding, purification, sterilization, shipping, and storage processes related to therapeutic recombinant protein products^1^. Several factors such as pH, temperature, shear force, protein and salt concentration, hydrophobic forces, and others may be responsible for protein instability^2^, potentially resulting in protein degradation, chemical modification such as oxidation and deamidation, disulfide bond formation or exchange, and aggregation. One of the most common issues for protein stability is aggregation^3^, for which various sizes and types of oligomers are possible, from relatively small oligomers such as dimers, trimers, and tetramers, to reversible and irreversible non-covalent oligomers involving chemical modification^4^. Sometimes, covalent oligomers may include intermolecular disulfide bonds, and one type of unfolded/misfolded state may lead others. Thus, it is very important to characterize protein instability under various conditions.

Although current standard immunomodulatory therapy with recombinant human IFN-β 1a (rhIFN-β 1a) has a favorable benefit-to-risk profile in terms of relapsing forms of multiple sclerosis (RRMS), clinical efficacy is potentially compromised due to the formation of neutralizing antibodies (NAbs) in patients^5–7^. Aggregation of rhIFN-β products has been proposed as a potential cause of neutralizing antibody formation^8–18^. In murine studies, an increase in IFN-β aggregates can induce immunogenicity^19,20^. In addition to aggregation, several studies also revealed various types of instability for IFN-β, including oxidation, deamidation, and disulfide changes, although their direct clinical relevance has not been confirmed^21,22^. These aggregated or unfolded/misfolded products can be induced during the production process and the product storage period due to the undesirable physicochemical properties of IFN-β. In particular, instability during production can affect both productivity and functional activity. Thus, IFN-β instability during the culture and purification, as well as during storage of the commercial product, has been investigated to decrease instability, especially aggregation, to improve productivity and maintain functional activity^23,1,24^.

In previous work, we developed the rhIFN-β 1a R27T mutant, and it exhibited improved biological activity^25^. This variant has a molecular weight of ~26 kDa with two N-glycosylation sites at amino acids 25 and 80. The additional glycosylation makes it more physicochemically stable and functionally effective, and increases the half-life. In particular, the R27T variant has higher thermostability than rhIFN-β 1a, due to added structural stabilization from additional glycosylation. However, like the native protein, there are stability issues for R27T production and storage due to hydrophobic amino acids. Additionally, there is a free thiol at Cys 17 and an intramolecular disulfide bridge between Cys 31 and Cys 141. The presence of a free cysteine in the R27T amino acid sequence is a concern regarding instability. Although we optimized culture and purification processes to stabilize the protein, minimize product-related impurities, and improve productivity, the production process involves various potential stresses such as shaking and shearing force, pH, temperature, and salt and protein concentration, any of which could alter the physical conformation, induce aggregation, or cause denaturation, chemical modification, and degradation. Aggregated or unfolded/misfolded states could affect the biological activity, immunogenicity, stability, and other characteristics of the R27T variant.

The purpose of the current study was to explore R27T instability during the production process, since this may affect final product stability. We investigated the stability state of R27T during optimal 50 L culture and subsequent purification. We also tested various stress conditions during culture and purification processes, and characterized the effects on instability. Under stressed conditions, we observed free thiol and disulfide bonding exchange without aggregation, revealing a new type of unfolded state, as well as aggregation and degradation, as occurs for rhIFN-β.

## Results

### R27T protein stability during culture

To explore the stability of R27T during the culture process, R27T-producing Chinese Hamster Ovary (CHO) cells were cultured under optimized 50 L bioreactor culture conditions. Table 1 shows cell growth, viability, and R27T production, and typical cell viability and production curves were obtained (Supplementary Fig. 1). Cell viability was maintained over 90% until the 7th day of culture, and cell viability was 88.7% at harvest, with a cell density of 7.39 × 10^6^ cells/mL at day 8 of culture. Total R27T production titer was confirmed by ELISA assay, and the functionally active R27T was measured by *in vitro* cytopathic effect (CPE) assay (Table 1). Both ELISA and CPE value have international unit (IU) as a unit. We found that the values (IU) from the ELISA and CPE was almost same when we conducted those assays with fully active substance such as Rebif and our in-house reference protein, R27T with 1.2 times small difference. ELISA value was 1.2 times higher than CPE value. That’s why it is necessary to use the correction factor 1.2 to calibrate values from ELISA and CPE when we compare those values. (data not shown). The native and unfolded R27T present during the culture process were quantified from the relative CPE/ELISA ratios and ELISA values minus CPE values, respectively, using an inter-assay correction factor of 1.2. As cultivation progressed, the relative native R27T ratio tended to increase, from 58.7% (day 5) to 93.1% (day 8). The absolute total amount of unfolded R27T tended to increase with elevated R27T production by CHO cells up to day 7, but interestingly, unfolded R27T tended to decrease by more than 3.5-fold at harvest (day 8). Although we cannot yet explain the recovery of a considerable proportion of inactive-to-active R27T by day 8, this tendency was also observed at the smaller 3 L scale, but not always (data not shown).

**Table 1:** 50 L Bioreactor culture profile for R27T production at 34 °C.

Culture Days	Viable cell (×10^6^ cells/mL)	Viability (%)	Total R27T	Active R27T		Unfolded R27T	Relative native R27T ratio
			ELISA (IU/mL)	CPE (IU/mL)	CPE × 1.2	50 L × {ELISA-(CPE × 1.2)} (IU)	CPE/ELISA ratio × 1.2 (%)
0	0.48	96.2	165,865 ± 10,137	98,452 ± 20,407	118,142	2,386,139,308	71.2
1	0.69	95.2	N.D.	N.D.	N.D.	N.D.	N.D.
2	0.91	95.2	N.D.	N.D.	N.D.	N.D.	N.D.
3	1.55	94.7	N.D.	N.D.	N.D.	N.D.	N.D.
4	2.46	95.3	N.D.	N.D.	N.D.	N.D.	N.D.
5	3.68	94.4	2,399,324 ± 150,893	1,173,529 ± 223,342	1,408,235	49,554,449,859	58.7
6	4.94	92.9	3,039,158 ± 174,828	1,639,658 ± 491,656	1,967,590	53,578,418,426	64.7
7	6.32	90.8	4,475,137 ± 386,245	2,416,502 ± 529,497	2,899,803	78,766,700,839	64.8
8	7.39	88.7	6,354,115 ± 468,134	4,930,157 ± 1,013,383	5,916,189	21,896,301,882	93.1

N.D., not detected.

### R27T protein stability during purification

R27T was purified from a 50 L culture supernatant using four optimized column steps after cell clarification, and each step was also analyzed by ELISA and CPE assays to estimate the relative native R27T ratio (Table 2). The cell clarification and affinity chromatography loading step in phosphate-based loading buffer under neutral conditions (pH 7.4) showed the lowest native R27T contents ratio (23.3–36.3%), but this was gradually recovered in subsequent affinity chromatography elution and ion exchange column steps. Although these steps were also conducted under neutral conditions (pH 7.0), this appeared to be compensated for by the propylene glycol (PG) additive, a well-known stabilizer as well as strong elution component for IFN-β. During the 3rd hydrophobic column step, the relative native R27T ratio decreased slightly, which might be due to the organic solvent, but the ratio was completely recovered during UF/DF and size exclusion column steps, after which R27T activity was shown 298.7 ± 18 MIU/mg full specific activity (data not shown). Thus, R27T was obtained in fully active form from the optimized purification steps.

**Table 2:** Titer and activity after different purification steps during R27T production.

Purification step	Buffer condition	Total R27T	Active R27T		Relative native R27T
		ELISA (IU/mL)	CPE (IU/mL)	CPE × 1.2	CPE/ELISA Ratio × 1.2 (%)
After cell clarification	pH 7.4, phosphate buffer	4,274,402 ± 52,611	1,293,531 ± 254,405	1,552,237	36.3
Affinity column (loading)	pH 7.4, phosphate buffer	2,554,198 ± 295,564	494,976 ± 67,158	593,971	23.3
Affinity column (elution)	pH 7.0, phosphate buffer, propylene glycol	47,143,890 ± 6,251,200	30,757,609 ± 2,304,273	36,909,131	78.3
Ion exchange column (loading)	pH2.9, phosphate buffer	N.D.	N.D.	N.D.	N.D.
Ion exchange column (elution)	pH 7.0, phosphate buffer, propylene glycol	53,179,005 ± 2,303,904	51,122,894 ± 7,757,284	61,347,473	115.4
Hydrophobic column (loading)	Acetonitrile based buffer	N.D.	N.D.	N.D.	N.D.
Hydrophobic column (elution)	Acetonitrile based buffer	56,184,296 ± 798,537	41,373,210 ± 12,196,181	49,647,852	88.4
UF/DF		N.D.	N.D.	N.D.	N.D.
Size exclusion column (loading)	Loading buffer	948,874,481 ± 56,818,547	859,683,700 ± 45,034,807	1,031,620,440	108.7
Size exclusion column (elution)	Final buffer	N.D.	N.D.	N.D.	N.D.
UF/DF purified R27T substance	Final buffer	283,399,550 ± 22,932,895	235,250,306 ± 18,704,365	282,300,367	99.6

N.D., not detected.

### R27T stability in the culture supernatant

**Figure 1:**
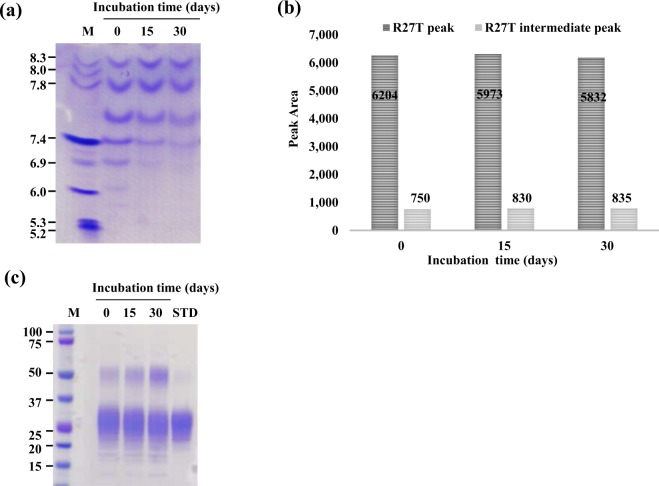
Stability of R27T in the culture supernatant. R27T was incubated in supernatant for 0, 15, and 30 days at the same temperature as the production process (15 °C) and partially purified by blue sepharose column chromatography for analysis. R27T physicochemical characteristics are shown for (**a**) IEF, (**b**) RP-HPLC (R27T peak: mainly double glycosylated target peak, R27T intermediate peak: single-glycosylation dominant R27T peak) and (**c**) SDS-PAGE under non-reducing conditions. In-house reference R27T was used for STD (standard).

The R27T culture supernatant obtained after cell clarification was stored for 0, 15, and 30 days at the same temperature as the production process (15 °C). After that, they are partially purified by affinity chromatography for analysis to investigate stability. During this time, some acidic or double-glycosylated R27T variants were degraded, according to the results of isoelectric focusing (IEF; Fig. 1(a)). More acidic or hydrophilic double-glycosylated R27T variants with abundant sialic acid were slightly decreased from 89.2% to 87.5%, while the single-glycosylation dominant R27T peak (intermediate peak) was increased from 10.8% to 12.5%, following reversed-phase high-performance liquid chromatography (RP-HPLC; Fig. 1(b)). Interestingly, upon degradation, the dimer was also increased over time, according to the results of sodium dodecyl sulfate–polyacrylamide gel electrophoresis (SDS-PAGE) under non-reducing conditions (Fig. 1(c)). To investigate the possible degradation effects by metalloenzymes, which are one of the main extracellular protease classes, in the culture supernatant during the typical process, EDTA was added to the culture supernatant at 100, 300, and 500 mM before purification (Supplementary Fig. 2). However, there was no large difference, indicating that there was no metalloenzymatic degradation during the routine production process.

### R27T stability under oxidative stress

**Figure 2:**
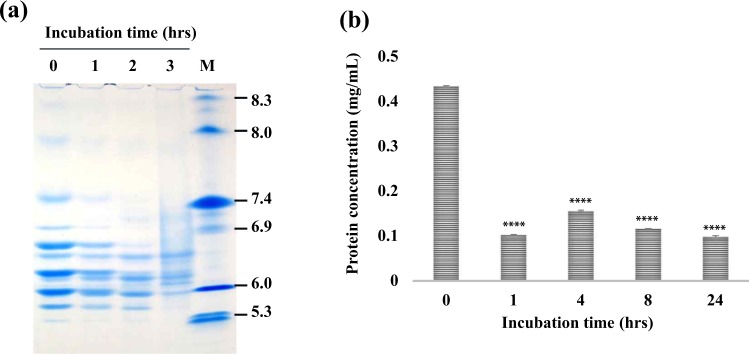
Evaluation of R27T stability under accelerated oxidation stress. (**a**) IEF; each lane 1, 2, 3, 4 refers to R27T incubation time with 0.05% H_2_O_2_ for 0, 1, 2, and 3 hrs, respectively. Lane 5: IEF marker. (**b**) R27T concentration was detected by Bradford assay after 0.05% H_2_O_2_ incubation for 0, 1, 4, 8, 24 hrs. The concentrations was presented as mean ± S.D. of three independent experiments. Each concentration was statistically analysed with 0 hrs concentration. ****p < 0.0001 was determined by one-way ANOVA, Dunnett’s multiple comparison tests.

We induced oxidative stress by incubating 433 μg/mL R27T with 0.05% hydrogen peroxidase (H_2_O_2_) for 24 h at 37 °C, and investigated physicochemical stability using IEF (Fig. 2(a)). We observed increased protein degradation with increasing H_2_O_2_ incubation time, especially for basic variants displaying single-glycosylation heterogeneity. Furthermore, Bradford assays confirmed protein degradation by revealing a decrease in protein concentration (Fig. 2(b)).

### R27T stability under temperature stress

**Figure 3:**
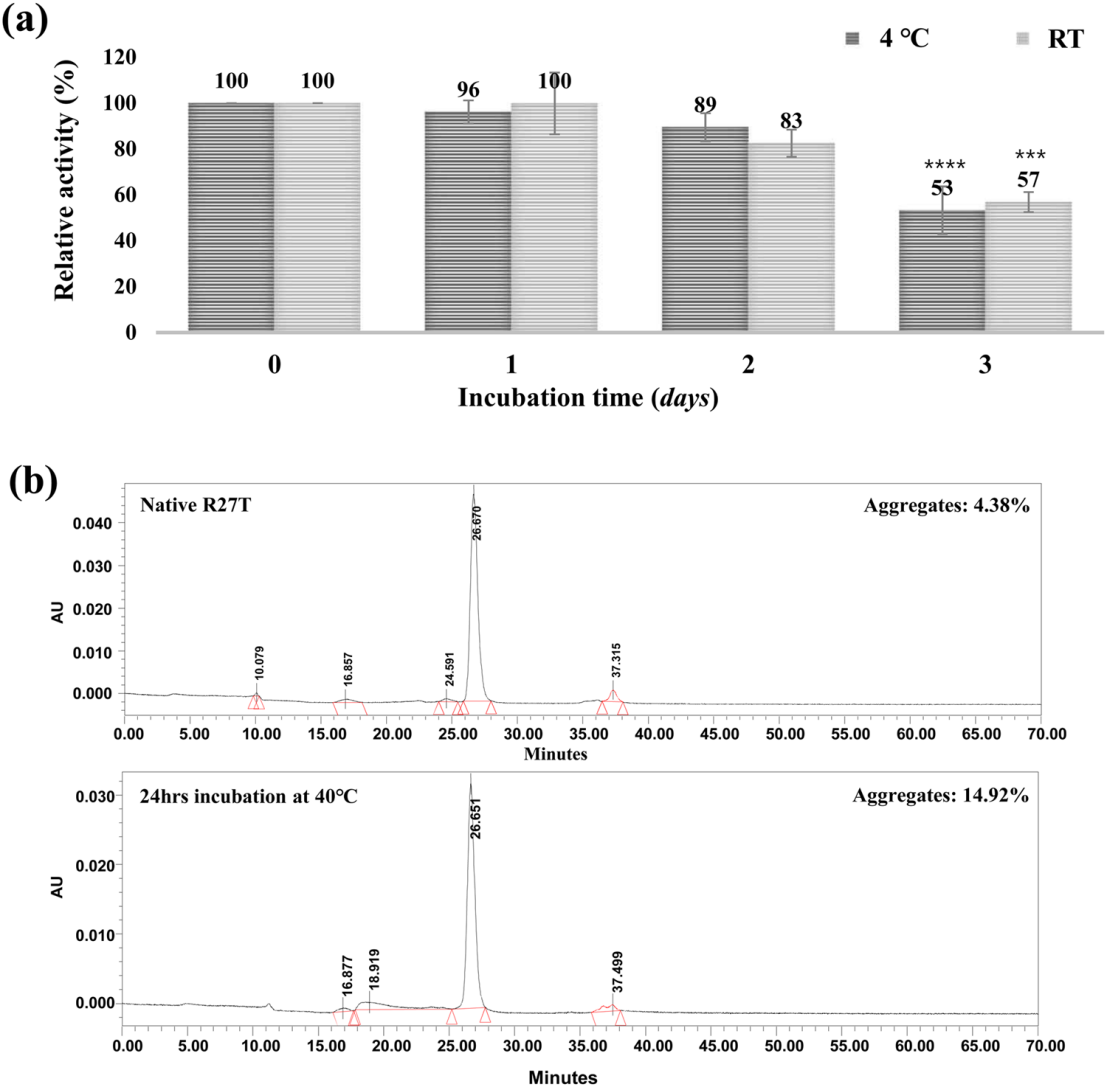
R27T stability under temperature stress during purification process. (**a**) Blue sepharose column eluate incubated at 4 °C and RT for 0, 1, 2, or 3 days. The biological activity was detected by CPE assay. The relative activity (%) was presented as mean ± S.D. of three independent experiments performed in triplicate. Each activity was statistically analysed with 0 hrs activity. ***p < 0.0005, ****p < 0.0001 was determined by one-way ANOVA, Tukey’s multiple comparison tests. (**b**) Purified R27T protein without formulation was incubated at 40 °C for 24 hr. R27T aggregation was analyzed by size exclusion chromatography.

Temperature is another type of stress that can often affect proteins during culture and purification. During the production process, R27T was incubated at RT overnight during the affinity step to allow a large amount of culture supernatant to be loaded. In this step, to evaluate temperature stress, the eluate from the affinity column was incubated at 4 °C and RT for 0, 1, 2, or 3 days (Fig. 3(a)). Functional activity analysis showed no change at either 4 °C or RT after 1 day, but activity began to decrease after this timepoint similarly at both 4 °C and RT. On day 3, R27T activity had decreased by up to one-third. Since affinity column step usually takes 12–24 hours, no stability problem can be considered to have occurred during normal affinity column process. We also tested the effect of higher temperature on purified R27T protein without formulation by incubating R27T at 40 °C for 24 h, and we investigated whether aggregation occurred by size exclusion chromatography (SEC). The results revealed a 10% increase in aggregation compared with the starting material (Fig. 3(b)).

### R27T stability under neutral pH stress

**Figure 4:**
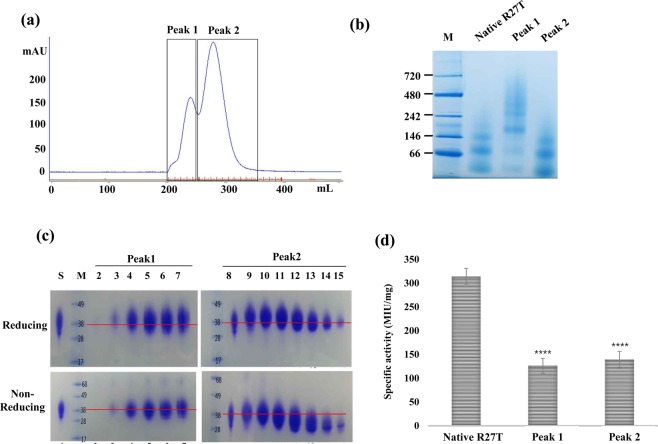
Effect of neutral pH stress and propylene glycol (PG) on R27T stability. The purification eluate from ion exchange column was incubated for 7 days at 4 °C, during which R27T was stored under elution conditions at pH 7.0 in phosphate-based buffer with 10% PG and 145 mM salt, and then purified by SEC for analysis. (**a**) The SEC chromatogram displayed an aggregation peak (peak 1) and a normal R27T peak (peak 2). (**b**) Aggregated R27T in peak 1 and normal R27T in peak 2 were also confirmed by native gel analysis. Active in-house reference R27T was used for native R27T. **(c**) Each SEC purification fraction was analysed by SDS-PAGE under reducing and non-reducing conditions. (**d**) Specific activity of collected peaks was investigated by CPE assays. The activity (MIU/mg) was presented as mean ± S.D. of three independent experiments performed in triplicate. Each activity was statistically analysed with native R27T activity. ****p < 0.0001 was determined by one-way ANOVA, Dunnett’s multiple comparison tests.

During the early purification steps including cellular clarification and Affinity and ion exchange columns, R27T was exposed to neutral conditions (pH 7.0−7.4) and contents of native R27T was relatively low in the absence of PG, as shown in Table 2. Thus, after the affinity column loading step, PG was added to stabilize the protein as described above. We investigated the effect of neutral pH with PG on R27T stability. The purification eluate from ion exchange column was incubated for 7 days at 4 °C, during which R27T was stored under elution conditions at pH 7.0 in phosphate-based buffer with 10% PG and 145 mM salt, and then purified by SEC for analysis. The SEC chromatogram displayed an aggregation peak (peak 1) and a normal R27T peak (peak 2; Fig. 4(a)). Aggregated R27T in peak 1 and normal R27T in peak 2 were also confirmed by native gel analysis (Fig. 4(b)). R27T in peak1 had a high molecular weight, while R27T in peak 2 was mainly monomeric with some oligomers, similar to active in-house reference R27T. We analyzed each SEC purification fraction by SDS-PAGE under reducing and non-reducing conditions (Supplementary Fig. 3, Fig. 4(c)). The most of R27T high-molecular-weight aggregates and some oligomers as shown in the native page gel (Fig. 4(b)) changed into monomer under non-reducing conditions, which confirmed the presence of almost no intermolecular disulfide-mediated aggregates (Fig. 4(c)). However, small amount of the disulfide bond mediated dimer was also observed. In addition, under reducing conditions, the proportion on monomer in each fraction increased slightly in apparent mass compared with non-reducing conditions, which was probably due to the breaking of the intramolecular disulfide bridge of R27T. One interesting point is that each non-reducing band from fractions of peak 2 displayed a greater decrease in apparent mass than bands from peak 1 under reducing conditions, indicating R27T misfolding, possibly due to changes in intra-disulfide bonds. Thus, we investigated specific activity to confirm the protein function of each peak (Fig. 4(d)). Interestingly, R27T in peak 2 that displayed a normal molecular mass in native gel analysis showed low specific activity, as did the aggregated form in peak 1, while the native R27T was fully functional with normal specific activity in the same oligomerization state. To better understand the effects of instability beyond simple aggregation on activity, we performed an in-depth analysis of low-activity non-aggregated R27T.

### Disulfide bond analysis of low-activity non-aggregated R27T

**Figure 5:**
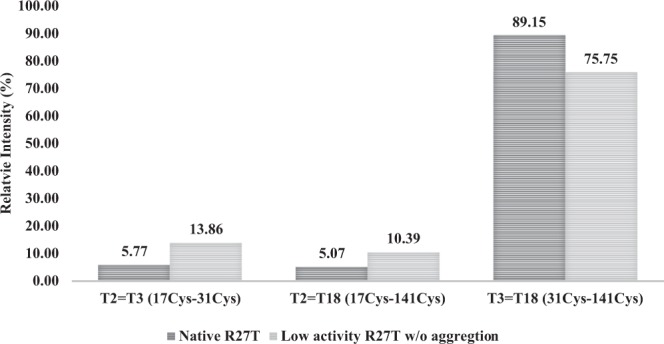
Disulfide bond analysis for native R27T and low-activity non-aggregated R27T. TOF-MS analysis was conducted with alkylated peptides from native R27T (active in-house reference R27T) and low-activity non-aggregated R27T. Quantitative analysis was performed by summing the extracted-ion chromatogram (XIC) area values according to the charge status of peptides.

We investigated whether intra-disulfide bond exchange occurred in low-activity non-aggregated R27T. To confirm the proper disulfide linkage pattern for R27T, namely a disulfide bond between Cys 31 and Cys 141, as well as a free thiol at Cys 17, Native R27T and low-activity non-aggregated forms of R27T were pretreated with alkylation, buffer-exchanged into TRIS-HCl, and digested with trypsin. Free thiols and disulfide bonds were predicted for T2 (Cys17), T3 (Cys31), and T18 (Cys141) fragments (Supplementary Fig. 4). Pretreated samples were analyzed by Triple time-of-flight (TOF) mass spectrometry (MS), and quantitative analysis was performed by summing the extracted-ion chromatogram (XIC) area values according to the charge status of peptides. Although the disulfide bond between T3 and T18 was abundant in both native R27T and low-activity non-aggregated R27T compared with other linkage, the disulfide bond between T3 and T18 in low-activity non-aggregated R27T (76%) showed much lower proportion than in native R27T (89%) (Fig. 5). The low-activity form also had more T2-T3 (14%) and T2-T18 (10%) disulfide bonds compared with native R27T (6% and 5%, respectively). Thus, low-activity non-aggregated R27T had more mismatched disulfide bonds than native R27T.

## Discussion

In this work, we investigated R27T stability during culture and purification processes to better understand the process-related instability of R27T under artificial stress conditions, to characterize the vulnerability of the protein to instability, and to define the critical instability state during the production process.

To monitor R27T stability during the production process, total R27T and functionally active R27T were estimated by ELISA and CPE assays, respectively. Among total R27T, relative native R27T ratio varied depending on the duration of the culture phase and purification steps. However, the ratio was recovered by the end of the overall production process, suggesting that culture and purification processes are optimized.

Although the unfolded proportion of R27T was recovered during the production process, transient unfolded states with low relative native R27T ratio were evident, indicating sub-optimal conditions. We therefore investigated the effects of different stresses, including stability within the supernatant, oxidative conditions, temperature stress, and neutral pH during production process. R27T in the supernatant may be susceptible to several factors such as host cell-derived proteases, media components, and storage temperature. In particular, enzymatic degradation may increase with decreases in cellular viability, which may result in the release of the enzyme from the dead cells used to express R27T. Thus, to minimize these phenomena, we harvested the supernatant when cell viability was >90% and stored the extract at 15 °C after filtration. In this study, supernatant incubation for 30 days at 15 °C as harsh condition, did indeed lead to degradation of R27T, especially R27T acidic variants, which may be desirable products with two glycosylation sites. R27T under supernatant stress conditions also formed dimers as well as degradation. Physical degradation may induce protein unfolding or misfolding, resulting in dimers. Oxidative stress can occur under certain dissolved oxygen (DO) conditions during bioreactor culture and exposure to air during purification processes^26^. Interestingly, R27T degradation under oxidative stress was evident for basic variants displaying single-glycosylation heterogeneity^27^. Temperature and neutral pH stresses mainly induced aggregation, which is well-known to destabilize rhIFN-β^9,21^. In particular, exposure to neutral pH during purification was highly damaging because it is very close to the isoelectric point of R27T (major pI ~5.3−6.90 and a minor pI 7.5, 8.0 and 8.3), and this resulted in lower specific activity and increased aggregation. Interestingly, some proportion of R27T after incubation under the neutral pH condition had the same apparent molecular mass as active in-house reference R27T but exhibited low activity corresponding to aggregated R27T. We therefore wondered what other factors may lower stability and activity without causing aggregation. We found that the folding state of R27T under reducing and non-reducing condition was different. Thus, we investigated intra-disulfide bond linkages, since they may cause incorrect folding and conformational changes, and rhIFN-β is known to contain some mismatched disulfide bonds^28^. In native R27T, more than 89% of molecules have a Cys31-141 disulfide bond, although a small fraction of native R27T molecules also have a mismatched disulfide bond between Cys17-141 or Cys17-31. Meanwhile, the low-activity non-aggregated form of R27T has a lower proportion of molecules (76%) with a Cys31-141 disulfide bond than native R27T, and more molecules have mismatched disulfide bond between Cys17-141 or Cys17-31. Our study showed that neutral pH stress could decrease functional activity and increase instability by increasing the proportion of the aggregated form and mismatched disulfide bonds in non-aggregated forms. Consistent with this observation, inhibition of mismatched disulfide bond formation by mutation of Cys-17 to serine has been shown to improve the physical stability of IFN-β^29^. Even in the absence of aggregation, misfolding due to intra-disulfide exchange can have immunogenic effects. Thus, intra-disulfide bond exchange without aggregation should be considered a novel type of unfolded state for the R27T variant, which can occur alongside traditional aggregation and degradation forms observed for native rhIFN-β. This is the first account of such an unfolded state for an IFN-β product.

## Materials and Methods

### R27T bioreactor cultures

A recombinant CHO cell line transfected with the gene encoding the human IFN- R27T variant was provided by ABION (Seoul, Korea). One stock vial was thawed and placed in 40 mL medium in a 125 mL Baffled Erlenmeyer flask, and was incubated at 37 °C with 5% CO_2_ and shaking at 100 rpm for 3 days. After 3 days, the cell density was measured, and 100 mL of culture at a density of 5.0 × 10^5^ cells/mL was added to a 250 mL flask and cultured for 2 days under the same conditions. Every 2 days, the cell density was measured and the culture was scaled up to 500 mL flasks, 1 L flasks, or a 7 L bioreactor. On the 3rd day of culture in a 7 L bioreactor, cells were cultured in a volume of 20 L in a 50 L culture bag for 1 to 2 days. An additional 30 L of medium was added to the 20 L seed culture fluid in which 200 mM L-glutamine was supplemented in a final volume of 50 L. The temperature was lowered to 34 °C and cultured at 30% DO, pH 7.2 ± 0.2, with shaking at 80 rpm. Harvesting was performed when cell viability was>85%.

### R27T purification process

On the 8th day of culture, 50 L culture solution was filtered through a 0.23 μm filter (3 M, MN, USA) to remove cell debris. The filtered culture supernatant was passed through a 0.2 μm sterile filter (Sartorius, Göttingen, Germany) and stored in a 50 L BPC bag (Thermo Scientific Hyclone, UT, USA) for purification. Purification of R27T was performed by four column steps. After cell clarification, the culture supernatant was applied to a blue sepharose 6FF affinity column (GE Healthcare, Buckinghamshire, UK), which was eluted at pH 7.0 with a PG-based phosphate buffer. Triple-diluted sepharose blue column eluate samples were loaded onto an ion-exchange column and eluted at pH 7.0 with phosphate buffer containing PG. The third column step was performed on a C4 RP-HPLC (Vydac, CA, USA). For the subsequent SEC step, the eluate was concentrated and filtered using a tangential flow filtration system (Millipore, MA, USA). A Sephacryl 100HR column was employed for gel filtration at a flow rate of 2.5 mL/min.

### R27T titer determination

The total amount of R27T was determined using a commercially available IFN‐β enzyme-linked immunosorbent assay (ELISA) kit (IBL, Hamburg, Germany) according to the manufacturer’s instructions. In brief, 96-well plates coated with goat anti‐human IFN‐β antibody were primed with 400 μL washing solution. After removing washing solution, diluted R27T samples and human IFN‐β standard solutions were mixed with horseradish peroxidase (HRP)‐labeled mouse monoclonal antibody in a microplate shaker, and were incubated at room temperature for 2 h. After three washes, color developer was added to each well, and plates were incubated for 30 min. The assay was terminated by addition of reaction stopper and the absorbance of the reaction mixture was measured at 450 nm using a plate reader.

### Active R27T determination (Cytopathic effect assay)

The amount of active R27T was determined based on antiviral activity using a cytopathic effect (CPE) assay. We used World Health Organization natural rhIFN (NIBSC code: 00/572) as a standard material. On the first day, A549 cells were mixed with serially diluted standard and R27T proteins in 96-well plates (triplicate were included for each sample). Triplicate wells for viral control was remained without cells. Plates were incubated for 20 h at 37 °C in a 5% CO_2_ incubator. The next day, encephalomyocarditis virus (EMCV, 1000 TCID/50 mL) was added and incubated for a further 22 h. Triplicate for cell control was remained without virus. On the third day, cells were dyed with Crystal Violet at room temperature for 1 h, excess dye was washed several times, samples were extracted with 2-methoxyethanol, and the absorbance at 570 nm was measured. R27T activity was determined by International Unit (IU), which was used to quantify biologically active substances. Standard material was served as a bioassay calibrant with 40,000 IU for measuring the potency of rhIFN. In this assay, IU was calculated as follows:

**Sample activity (IU/ml) =Standard activity (IU/ml) of 1** ^**st**^**row X 2** ^**(Nsam-Nstd)**^**X Dilution fold number of 1** ^**st**^**row sample**

**(Ac: absorbance mean value of cell control, Vc: absorbance mean value of virus control, A**_**50**_**: (Ac** + **Vc)/2, Nstd: n** + **(A**_**n**_**-A**_**50**_**)/(A**_**n**_**-A**_**n+1**_**), Nsam: n** + **(A**_**n**_**-A**_**50**_**)/(A**_**n**_**-A**_**n+1**_**), N: exponent of dilution times (2**^**n**^**) correspond to the lowest OD value among high OD value more than A**_**50**_**)**

### UV spectroscopy

A 500 µL sample of 0.5 M NaOH and a 500 µL test sample were mixed in a coated microtube. A blank sample comprising 50 μL of 0.5 M NaOH and 50 μL test sample buffer was also prepared. Each mixture was centrifuged, the blank sample was used for zeroing, and UV absorbance was measured at a wavelength of 290 nm on an Agilent Cary 100UV/VIS spectrophotometer in a quartz cuvette with a path length of 1 cm. The absorbance of each sample was measured in triplicate.

### Preparation of unfolded/misfolded R27T under stress conditions

To investigate the stability of R27T within the supernatant, the R27T culture product was incubated for 0, 15, or 30 days at 15 °C following cell clarification after small-scale culture. After incubation, each sample was purified by blue sepharose column chromatography for further analysis. To probe the effect of enzyme degradation in the supernatant, EDTA was added at a concentration of 100, 300, or 500 mM. To obtain oxidized R27T, untreated 433 μg /mL R27T was incubated with 0.05% hydrogen peroxidase (H_2_O_2_) for 1, 2, 3, or 24 h at 37 °C. The reaction was stopped by adding 100 mM EDTA to a final concentration of 1 mM. To determine the protein concentration in the reacted samples, a micro Bradford protein assay (Thermo Fisher Scientific, USA) was performed according to the manufacturer’s instructions. To test temperature stress, the blue sepharose column elute sample was incubated at 4 °C or RT for 1, 2, 3, or 4 days. To investigate more extreme thermal stability, purified R27T was incubated at 40 °C for 24 h. Neutral pH was employed as the final stress condition. The partially purified intermediate product obtained after ion-exchange chromatography was incubated for 7 days at 4 °C, and eluates were stored at pH7.0 in phosphate-based buffer containing PG.

### SDS-PAGE and western blotting

Pre-cast NuPAGE 4–12% BIS-TRIS gels (Invitrogen) were run under non-reducing (100 °C, 2 min boiling) and reducing (2 μL β-mer, 100 °C, 2 min boiling) conditions for SDS-PAGE and western blotting. A 5–7 μg sample was loaded each time and run at 200 V for 30 min at room temperature. For SDS-PAGE, Coomassie staining was performed. After SDS-PAGE, western blotting was performed by transfer at 100 V for 1 h and blocking with 5% skim milk at RT overnight. Mouse anti-human IFN-β (1:5000) primary antibody was incubated at RT for 1 h, and goat anti-mouse IgG-HRP (1:2000) secondary antibody was incubated at RT for 1 h. For native page, a 3–12% gel was used, and all other steps were performed as described for SDS-PAGE.

### RP-HPLC

For RP-HPLC, samples were analyzed with a Vydac C4 column (250 × 4.6 mm). Chromatograms were recorded using a 1260 MWD VL UV detector, an Agilent 1260 Infinity instrument, and a 1260 ALS autosampler operating at a flow rate of 1 mL/min. Mobile phase A buffer consisted of 0.1% trifluoroacetic acid (TFA; Sigma) in 100% distilled water, and buffer B consisted of 100% acetonitrile (Merck) and 0.1% TFA (Sigma).

### HP-SEC

For HP-SEC, samples were analyzed with a TSKgel G3000SWxL column (7.8 × 300 mm, 5 μm). Chromatograms were recorded with a 1260 MWD VL UV detector, an Agilent 1260 Infinity instrument, and a 1260 ALS autosampler operating at a flow rate of 0.8 mL/min. The mobile phase consisted of 100 mM sodium phosphate dibasic buffer, 150 mM sodium chloride, and 0.05% (w/v) SDS at a pH of 7.0, and was filtered through a 0.2 μm filter prior to use.

### IEF

For IEF, Novex IEF Cathode buffer (10×, pH 3–10) was employed. R27T was mixed with 2× sample buffer. After sample preparation, samples were subjected to 100 V for 1 h, 200 V for 1 h, or 500 V for 30 min. After this, the gel was incubated with 12% trichloroacetic acid (TCA) buffer for 30 min with shaking. After removing 12% TCA buffer, the gel was shaken in distilled water for 10 min, stained with Coomassie Instant Blue while shaking for 30 min to 1 h, and then destained for 1–2 h with distilled water.

### Disulfide bond linkage analysis

The alkylating agent iodoacetic acid (IAA) was added to the sample, mixed, and reacted at room temperature in the dark for 60 min. After reaction, samples were mixed with denaturing buffer (7 M guanidine HCl, 1 mM EDTA, 0.36 M TRIS-HCl, pH 8.6) and incubated at room temperature for 60 min. The sample was then desalted using a filter tube and exchanged into 50 mM TRIS-HCl pH 7.8. A 50 μL volume of buffer-exchanged sample solution was mixed with 1 μL Peptide:N-glycosidase F (PNGase F) and reacted for 10 min at 400 W and 37 °C using REDS. A 1 μL sample of 1 mg/mL trypsin was added and reacted at 37 °C for 18 h. The reaction was terminated using 1 μL formic acid, and analysis was performed using a LC mass spectrometer. The peptides were separated by reverse phase UPLC equipped with a 2.1 mm × 100 mm, 130 Å BEH C18, Waters column using an increasing gradient of acetonitrile in water. The peptides were identified by on-line mass spectrometry (LC-MS/MS) using Triple Q-TOF 5600 + , AB sciex Mass spectrometry.

### Statistical analysis

All values are presented as means ± standard deviation (S.D.). Where indicated, significance was analyzed using one-way analysis of variance (ANOVA) with appropriate Dunnett’s or Tukey’s test analysis for multiple group. Significance was defined at ***p < 0.0005, ****p < 0.0001 using GraphPad Prism 7.0 software.

## Supplementary information

Supplementary information.
