# Supplementary material for: Stability and Activity of the Hyperglycosylated Human Interferon-β R27T Variant
Source: Sci Rep. 2020 May 21;10:8412. doi: 10.1038/s41598-020-65495-y (PMC7242330; doi:10.1038/s41598-020-65495-y)
Supplement: Supplementary file 1 — Supplementary information. [file 41598_2020_65495_MOESM1_ESM.pdf]

Stability and Activity of the Hyperglycosylated Human Interferon-β R27T Variant

- Supplementary Information

Kyoung Song [1*], Dae Bong Moon [2], Na Young Kim [3], Young Kee Shin [4,5,6*]

[1]*Center for Companion Diagnostics, LOGONE Bio Convergence Research Foundation, Seoul, Republic of Korea*

[2]*IFEZ Bio Analysis Center, Binex Co., Ltd., Incheon, Republic of Korea*

[3]*ABION Inc., R&D Center, Seoul, Republic of Korea*

[4]*Research Institute of Pharmaceutical Sciences, College of Pharmacy, Seoul National University, Seoul, Republic of Korea*

[5]*Molecular Medicine and Biopharmaceutical Sciences, Graduate School of Convergence Science and Technology, Seoul National University, Seoul, Republic of Korea*

[6]*Bio-MAX/N-Bio, Seoul National University, Seoul, Republic of Korea*

*** Corresponding Authors:**

Kyoung Song, Ph.D.

Center for Companion Diagnostics, LOGONE Bio Convergence Research Foundation, Seoul, Republic of Korea

Telephone: +82-2-880-9187

E-mail: sk17@logonebio.org

Young Kee Shin, M.D., Ph.D.

Research Institute of Pharmaceutical Sciences, College of Pharmacy, Seoul National University, Seoul, Republic of Korea

Molecular Medicine and Biopharmaceutical Sciences, Graduate School of Convergence Science and Technology, Seoul National University, Seoul, Republic of Korea

Bio-MAX/N-Bio, Seoul National University, Seoul, Republic of Korea

Telephone: +82-2-880-9187

E-mail: ykeeshin@snu.ac.kr

Supplementary Figure 1.

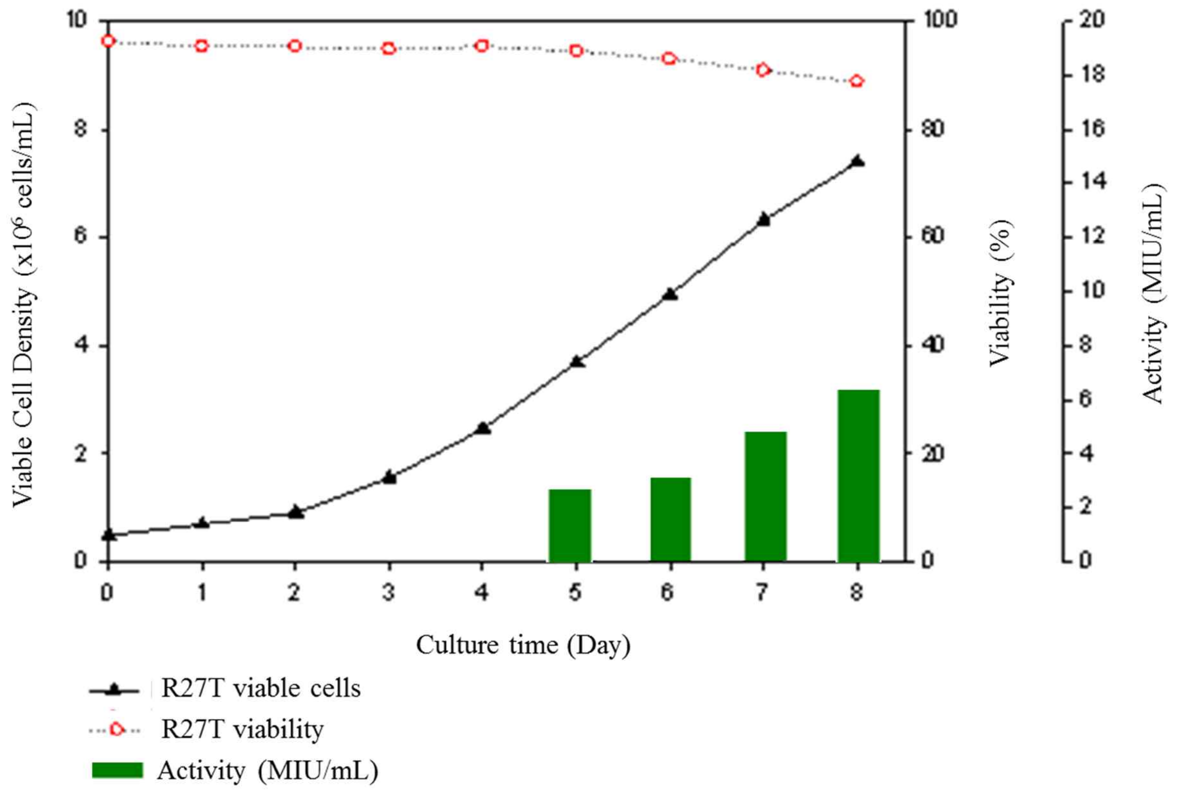

50 L bioreactor profile for R27T production by CHO cells.

Supplementary Figure 2.

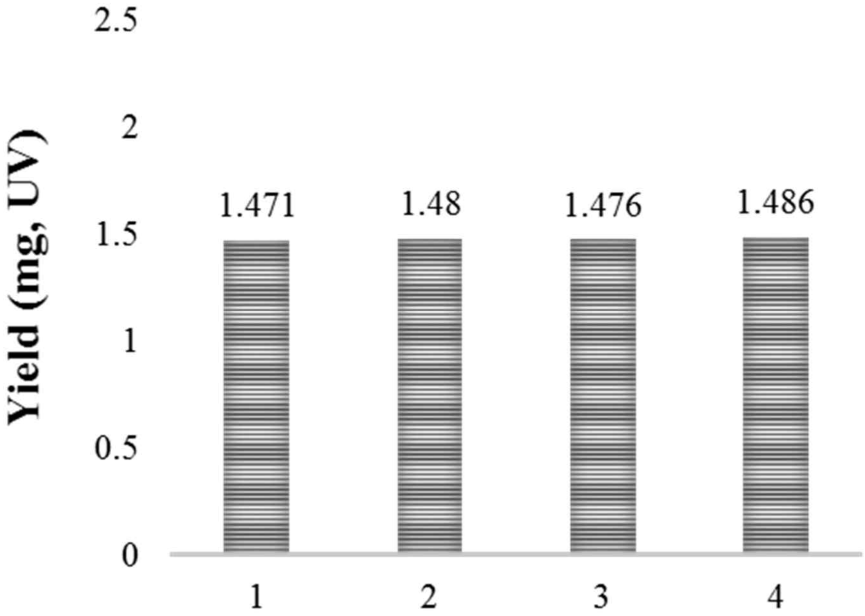

Analysis of enzymatic degradation in the culture supernatant using EDTA. R27T concentration was measured by UV spectrometer after affinity chromatography. Lane 1, supernatant containing R27T; Lane 2, supernatant with 100 mM EDTA; Lane 3, supernatant with 300 mM EDTA; Lane 4, supernatant with 500 mM EDTA.

Supplementary Figure 3.

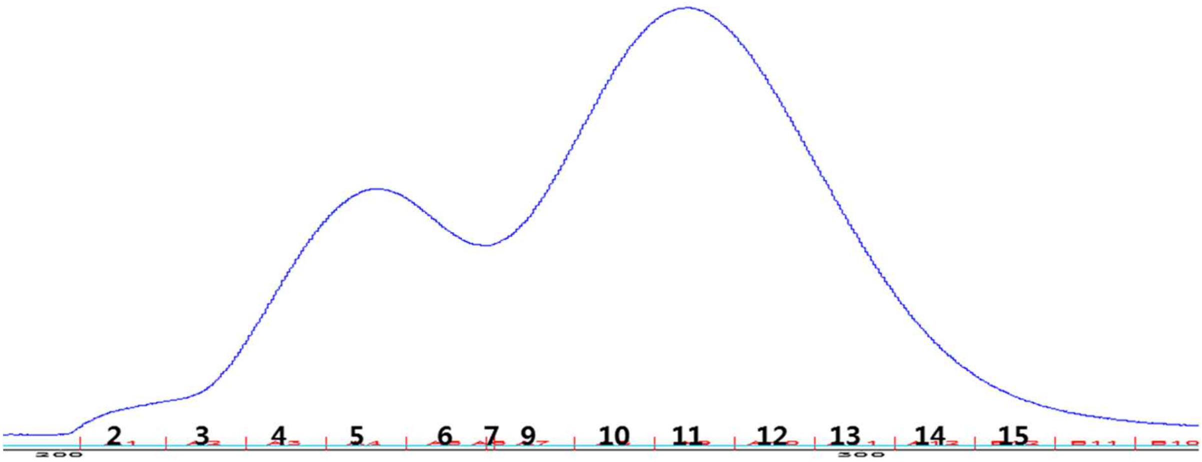

Size exclusion chromatogram of R27T under neutral pH stress.

Supplementary Figure 4.

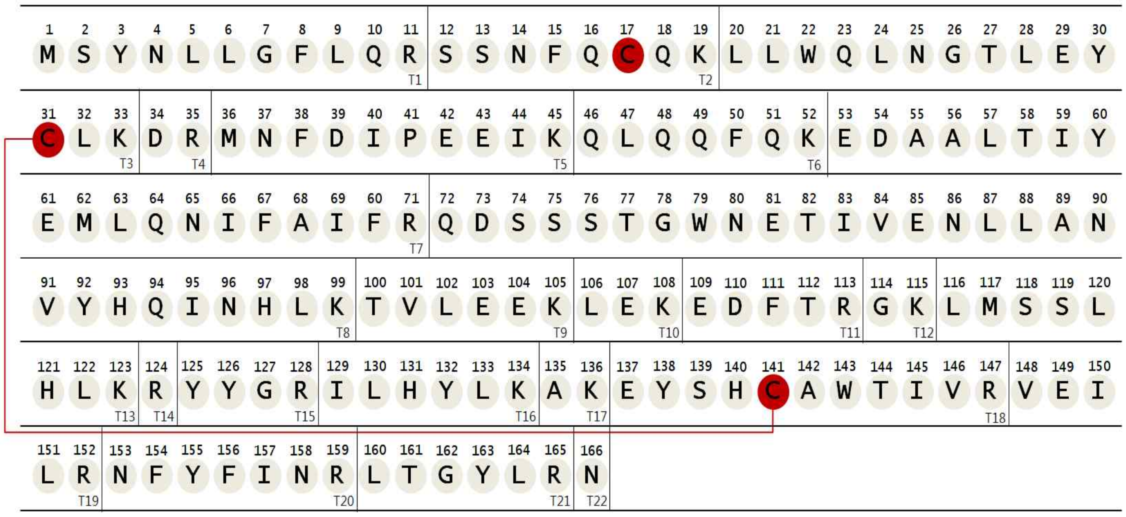

Prediction of the amino acid sequence of R27T fragments generated by trypsin digestion.
